# Kikuchi-Fujimoto disease

**DOI:** 10.1186/1750-1172-1-18

**Published:** 2006-05-23

**Authors:** Xavier Bosch, Antonio Guilabert

**Affiliations:** 1 Department of Internal Medicine, Hospital Clinic, University of Barcelona, Spain; 2 Department of Dermatology, Hospital Clinic, University of Barcelona, Spain

## Abstract

Kikuchi-Fujimoto disease (KFD) is a benign and self-limited disorder, characterized by regional cervical lymphadenopathy with tenderness, usually accompanied with mild fever and night sweats. Less frequent symptoms include weight loss, nausea, vomiting, sore throat. Kikuchi-Fujimoto disease is an extremely rare disease known to have a worldwide distribution with higher prevalence among Japanese and other Asiatic individuals. The clinical, histopathological and immunohistochemical features appear to point to a viral etiology, a hypothesis that still has not been proven. KFD is generally diagnosed on the basis of an excisional biopsy of affected lymph nodes. Its recognition is crucial especially because this disease can be mistaken for systemic lupus erythematosus, malignant lymphoma or even, though rarely, for adenocarcinoma. Clinicians' and pathologists' awareness of this disorder may help prevent misdiagnsois and inappropriate treatment. The diagnosis of KFD merits active consideration in any nodal biopsy showing fragmentation, necrosis and karyorrhexis, especially in young individuals presenting with posterior cervical lymphadenopathy. Treatment is symptomatic (analgesics-antipyretics, non-steroidal anti-inflammatory drugs and, rarely, corticosteroids). Spontaneous recovery occurs in 1 to 4 months. Patients with Kikuchi-Fujimoto disease should be followed-up for several years to survey the possibility of the development of systemic lupus erythematosus.

## Disease name and synonyms

Kikuchi-Fujimoto disease

Kikuchi's disease

Histiocytic necrotizing lymphadenitis

## Definition

Kikuchi-Fujimoto disease (KFD) is an enigmatic, benign and self-limited syndrome characterized by regional lymphadenopathy with tenderness, predominantly in the cervical region, usually accompanied by mild fever and night sweats.

Initially described in Japan, KFD was first reported in 1972 almost simultaneously by Kikuchi [1] and by Fujimoto *et al*. [2] as a lymphadenitis with focal proliferation of reticular cells accompanied by numerous histiocytes and extensive nuclear debris [3].

## Epidemiology

Kikuchi-Fujimoto disease is an extremely rare disease known to have a worldwide distribution with a higher prevalence among Japanese and other Asiatic individuals [4]. Only isolated cases are reported in Europe. Affected patients are most often young adults under the age of 30 years; the disease is seldom reported in children. A female preponderance of cases has been underlined in the literature (female to male ratio 4:1). Recent reports seem to indicate that the female preponderance was overemphasized in the past and that the actual ratio is closer to 1:1 [4,5].

## Etiology and pathogenesis

There is much speculation about the etiology of KFD. A viral or autoimmune cause has been suggested. The role of Epstein-Barr virus, as well as other viruses (HHV6, HHV8, parvovirus B19) in the pathogenesis of KFD remains controversial and not convincingly demonstrated [4]. A viral infection is, nonetheless, possible by virtue of clinical manifestations, as described by Unger *et al*. [6] that include upper respiratory prodrome, atypical lymphocytosis and lack of response to antibiotic therapy, and certain histopathologic features (*i.e*., T-cells as revealed by immunological marker studies). KFD has also been recorded in HIV- and HTLV-1-positive patients [7].

On the other hand, electron microscopic studies have identified tubular reticular structures in the cytoplasm of stimulated lymphocytes and histiocytes in patients with KFD [3]. Since these structures have also been noted within endothelial cells and lymphocytes of patients with systemic lupus erythematosus (SLE) and other autoimmune disorders, some authors hypothesized that KFD may reflect a self-limited autoimmune condition induced by virus-infected transformed lymphocytes [8]. It is possible that KFD may represent an exuberant T-cell mediated immune response in a genetically susceptible individual to a variety of non-specific stimuli [4].

Although the mechanism of cell death involved in KFD has not been extensively studied, Ohshima *et al*. have shown that apoptotic cell death may play a role in the pathogenesis of the disease [9]. According to these authors, proliferating CD8 positive T-cells may act as "killers" and "victims" in the apoptotic process *via *Fas- and perforine- pathways.

## Clinical manifestations

The onset of KFD is acute or subacute, evolving over a period of two to three weeks. Cervical lymphadenopathy is almost always present consisting of tender lymph nodes that involve mainly the posterior cervical triangle. Lymph node size has been found to range from 0.5 to 4 cm, but it may reach 5 to 6 cm and rarely larger than 6 cm. Generalized lymphadenopathy can occur [5,10] but is very rare. In addition to lymphadenopathy, 30 to 50% of patients with KFD may have fever, usually of low-grade, associated with upper respiratory symptoms. Less frequent symptoms include weight loss, nausea, vomiting, sore throat and night sweats [11,12]. Leukopenia can be found in up to 50% of the cases. Atypical lymphocytes in the peripheral blood have also been observed. Involvement of extranodal sites in KFD is uncommon but skin, eye and bone marrow affection, and liver dysfunction have been reported [4]. KFD has also been reported as a cause of prolonged fever of unknown origin [13]. Although the disease has been linked to SLE, as well as to other autoimmune conditions [4,14], the real strength of such associations remains to be clarified. There have been anecdotal reports of unusual features of KFD including carcinoma [15], diffuse large B-cell lymphoma [16] and hemophagocytic syndrome [17]. There are occasional reports describing cases of extranodal skin involvement or, even more rarely, of fatal multicentric disease.

## Diagnosis

Kikuchi-Fujimoto disease is generally diagnosed on the basis of an excisional biopsy of affected lymph nodes. No specific diagnostic laboratory tests are available. The results of a wide range of laboratory studies are usually normal. Nevertheless, some patients have anemia, slight elevation of the erythrocyte sedimentation rate and even leukopenia. Of note, one third of patients present atypical peripheral blood lymphocytes [5]. Characteristic histopathologic findings of KFD include irregular paracortical areas of coagulative necrosis with abundant karyorrhectic debris, which can distort the nodal architecture, and large number of different types of histiocytes at the margin of the necrotic areas. The karyorrhectic foci are formed by different cellular types, predominantly histiocytes and plasmacytoid monocytes but also immunoblasts and small and large lymphocytes. Neutrophils are characteristically absent and plasma cells are either absent or scarce. Importantly, atypia in the reactive immunoblastic component is not uncommon and can be mistaken for lymphoma [18]. The immunophenotype of KFD typically consists of a predominance of T-cells, with very few B-cells. There is an abundance of CD8+ T-cells over CD4+. The histiocytes express histiocyte-associated antigens such as lysozyme, myeloperoxidase (MPO) and CD68.

Finally, striking plasmacytoid monocytes are also positive for CD68 but not for MPO [4].

## Differential diagnosis

Although KFD is considered very uncommon, this disorder must be included in the differential diagnosis of 'lymph node enlargement' since its course and treatment differ dramatically from those of lymphoma, tuberculosis and SLE. The histological differential diagnosis of KFD mainly includes reactive lesions as lymphadenitis associated with SLE or herpes simplex, non-Hodgkin's lymphoma, plasmacytoid T-cell leukemia, Kawasaki's disease, myeloid tumor and even metastasic adenocarcinoma [4].

The differentiation of KFD from SLE can sometimes be problematic because both can show similar clinical and histological features. Furthermore, KFD has been reported in association with SLE. In this case, before making the diagnosis of KFD, laboratory tests including C3, C4, anti-Sm, and LE cells are needed to rule out SLE.

The diagnosis of KFD is generally not difficult, although early lesions lacking overt necrosis can be misdiagnosed as malignant lymphoma, due to the presence of abundant immunoblasts [7]. Features of KFD that may help prevent its misdiagnosis as malignant lymphoma include incomplete architectural effacement with patent sinuses, presence of numerous reactive histiocytes, relatively low mitotic rates, absence of Reed-Sternberg cells.

The recognition of KFD is necessary because one can avoid laborious investigation for infectious and lymphoproliferative diseases.

## Clinical course and management

Kikuchi-Fujimoto disease is typically self-limited within one to four months. A low but possible recurrence rate of 3 to 4% has been reported [3]. In some few patients, SLE may occur some years later. No risk to other family members is felt to be associated with KFD [7]. Symptomatic measures aimed to relief the distressing local and systemic complains should be employed. Analgesics-antipyretics and nonsteroidal anti-inflammatory drugs may be used to alleviate lymph node tenderness and fever. The use of corticosteroids has been recommended in severe extranodal or generalized KFD but is of uncertain efficacy. Surgical consultation may be indicated for a diagnostic excisional lymph node biopsy. Patients with KFD require a systematic survey and regular follow-up for several years to rule out the development of SLE. The cervical lymphadenopathy runs a benign course and appears to resolve spontaneously 1 to 6 months after definite diagnosis.
